# Discovery of an Anteriorly Deviated, Partially Ossified Xiphoid Process With a Large, Teardrop-Shaped Foramen in a Male Cadaver

**DOI:** 10.7759/cureus.61068

**Published:** 2024-05-25

**Authors:** Michelle Sue, Peter Lombardi, Annie Shi Ru Li, Harun Bola, Danielle C Bentley

**Affiliations:** 1 Faculty of Arts & Science, University of Toronto, Toronto, CAN; 2 Department of Surgery, Division of Anatomy, University of Toronto, Toronto, CAN

**Keywords:** variations, embryology, xiphoid foramina, xiphoid process, anterior thoracic wall, xiphoid process foramen, sternal variations

## Abstract

The sternum, or “breastbone,” is a principal bony component of the anterior thoracic wall and comprises the manubrium of the sternum, the body of the sternum, and the xiphoid process. The xiphoid process is the most inferior of these elements and commonly presents as a small, solid bone shaped like an inverted triangle. However, clinical literature has reported numerous variations in its size, shape, and presentation, likely the result of its lengthy embryological development from cartilage into fully ossified bone. In this case report, a rare, anteriorly deviated, partially ossified xiphoid process with a large, teardrop-shaped foramen is presented that was discovered during a routine cadaveric dissection of a 75-year-old male within an undergraduate anatomy course. Although anatomical variations in the xiphoid process are often asymptomatic and often only found incidentally through CT or X-ray scans, healthcare professionals should be aware of such variations to avoid both misdiagnoses as well as iatrogenic complications.

## Introduction

Along the midline of the anterior thoracic wall lies the sternum, a broad, flat bone comprising three elements: the superiorly located manubrium of the sternum, followed by the body of the sternum in the middle, and the inferiorly located xiphoid process [1,2]. Structurally, the xiphoid process is a small, solid bone, shaped like an inverted triangle with its apex extending into the epigastrium region of the abdomen [1,3]. Visually, the bone resembles the tip of a sword, aligning with its Greek etymological origin, where the term “xiphoid” is derived from *xiphos*, meaning “straight sword” [4]. Functionally, the xiphoid process contributes to the border of the inferior thoracic aperture while serving as an attachment point for the linea alba inferiorly, the diaphragm muscle posteriorly, and the most medial fibers of the rectus abdominis muscle and the aponeuroses of the internal and external oblique muscles anteriorly [1]. An atypical xiphoid process may potentially disrupt these aponeurotic and muscular attachments [1].

Formation of the sternum arises from numerous prenatal and postnatal ossification centers, with recent literature recording up to six centers: one within the manubrium of the sternum, up to four throughout the body of the sternum, and one within the xiphoid process [5,6]. Due to this highly multifarious development, anatomical variations throughout the sternum have been thoroughly documented and most commonly include manubriosternal fusion, sternoxiphoidal fusion, a sternal cleft or defect, a sternal foramen, and/or a suprasternal tubercle [7]. Of particular interest, although the xiphoid process begins its cartilaginous development prenatally along with the manubrium and body of the sternum, it only begins to slowly ossify from this cartilaginous precursor in the 1st decade of life [6]. Ossification of the xiphoid process is typically completed between the 3rd and 4th decade of life, marked by the formation of a synostosis at the xiphisternal joint [2,8,9]. However, primary literature has also reported instances of predominantly cartilaginous xiphoid processes in patients as old as 80 years (corresponding to the end of the 7th decade of life) [10]. Given the xiphoid process’s lengthy developmental time, the literature has reported numerous variations in xiphoid process morphology due to erroneous development or fusion of the ossification center, including bifid, trifid, curved, deflected, and xiphoid process foramina [2,5,7,11]. 

Although many sternal structural variations are asymptomatic and benign, an appreciation of such variations is of great importance to healthcare professionals, especially in radiological or clinical settings. Their incidental, unexpected discovery may result in a misdiagnosis due to misinterpretation of a novel sternal variation. For example, in bone scintigraphy, photopenic areas due to sternal foramina have previously been mistaken for osteolytic lesions [12]. Moreover, an anteriorly deviated xiphoid process may be mistaken for an epigastric mass and, in some cases, trigger xiphodynia from inflammation and irritation of the local area [13]. As such, it is paramount for healthcare professionals to be aware of both sternal and xiphoid variations in order to ensure accurate interpretations of both radiological images and physical examinations that lead to appropriate diagnoses and associated treatment plans.

## Case presentation

During a routine educational cadaveric dissection in an advanced undergraduate class at the University of Toronto, an anteriorly deviated xiphoid process with a significant foramen was uncovered. The formalin-fixed donor was an elderly 75-year-old male. The reported cause of death was chronic obstructive pulmonary disease (COPD), and there were no signs of past surgical intervention or trauma to the thoracic area. Students followed Grant’s Dissector (17th edition) for in-laboratory dissection tasks [14]. 

**Figure 1:**
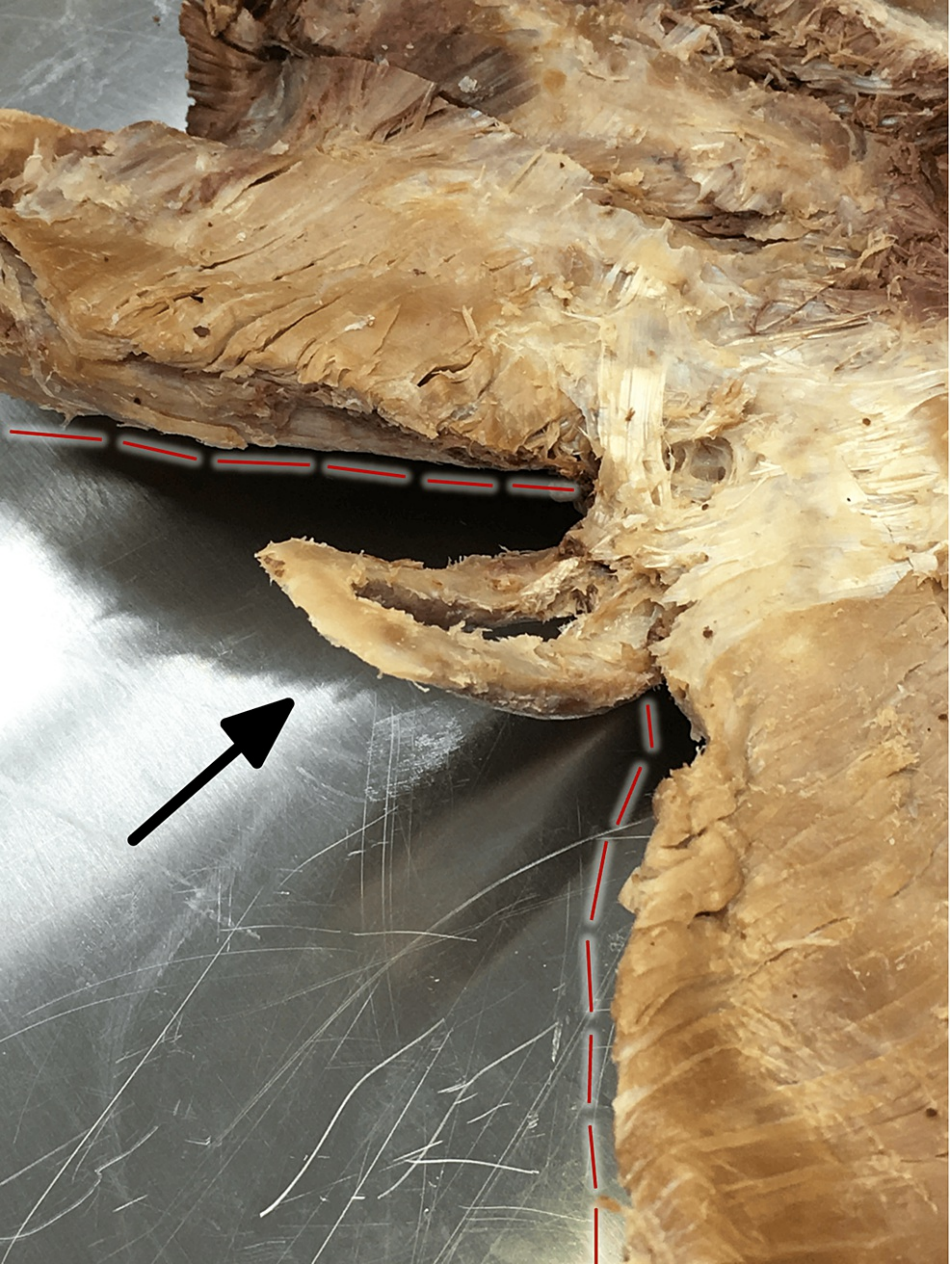
Anterior aspect of the sternum The anterior aspect of the sternum (oblique angle), showing the medial aspect of the costal margin (with adjacent red dashed lines) and an anteriorly deviated xiphoid process (arrow) with a foramen.

While following standard instructions for the dissection of the anterior thoracic wall, bilateral projections extending inferiorly from the sternoxiphoid joint at the base of the body of the sternum were initially uncovered. It was observed that these inferior projections were also anterior deviated. Examination of the area continued under the suspicion that the donor may have had a bifid xiphoid process. However, upon further investigation and careful dissection of surrounding soft tissues, it was discovered that the initial, proximal bifurcation joined back together inferiorly, encompassing a complete xiphoid foramen (Figure 1).

**Figure 2:**
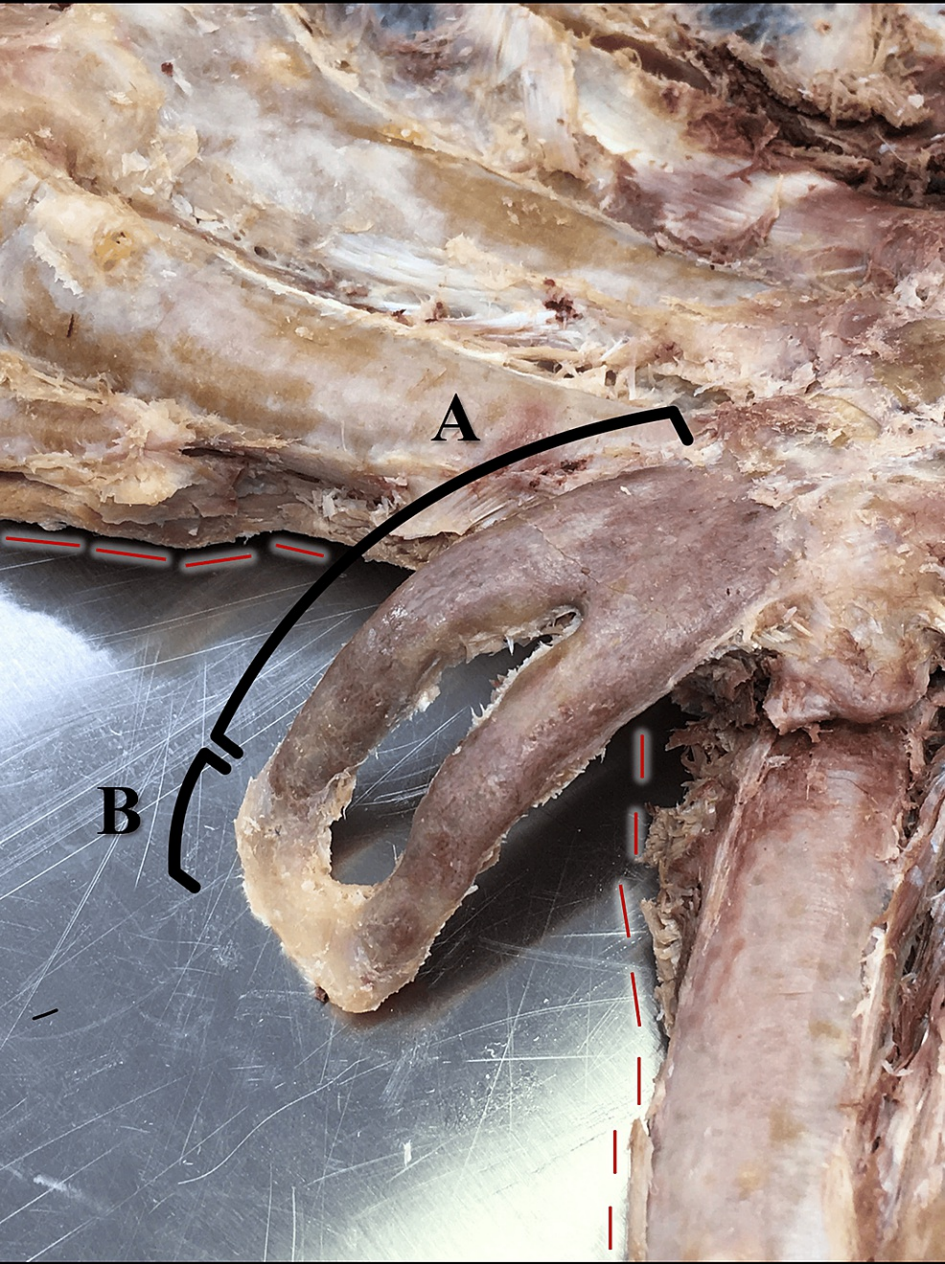
Posterior aspect of the sternum Posterior aspect of the sternum (oblique angle), focused on the xiphoid process with a prominent xiphoid foramen. The xiphoid process is partially ossified superiorly (A) but appears cartilaginous inferiorly (B). Red dashed lines indicate the medial aspect of the costal margin.

Although histological samples were not taken, gross inspection of the structure revealed that the superior boundaries of the xiphoid foramen were fully ossified, while the inferior boundaries remained cartilaginous (Figure 2). The foramen itself had a length of 1.2 cm and was teardrop-shaped: narrow superiorly and broad inferiorly with a maximum width of 0.8 cm (Table 1).

**Table 1:** Measurements of the specimen

Sternal section	Measurement
Length of the entire sternum	21.5 cm (8.5 in)
Length of manubrium	4.9 cm (1.9 in)
Maximum width of manubrium	5.8 cm (2.3 in)
Length of sternum from sternal angle to tip of xiphoid process	16.9 cm (6.7 in)
Length of the body of the sternum	12.0cm (4.7 in)
Length of xiphoid process	4.3 cm (1.7 in)
Breadth of the xiphoid process	2.2 cm (0.9 in)
Length of xiphoid process foramen	2.9 cm (1.1 in)
Maximum width of xiphoid process foramen	0.8 cm (0.3 in)

## Discussion

This clinical case report has described a rare, anteriorly deviated, partially ossified xiphoid process with a large, teardrop-shaped foramen. An atypical xiphoid process such as this may be the result of early abnormalities in sternal development. Embryologically, the sternum is derived from lateral plate mesoderm that migrates to form two longitudinal mesenchymal bands in the ventrolateral body wall, also known as sternal bars [2]. The lateral aspect of each sternal bar joins the upper seven ipsilateral costal cartilages, while the medial aspects fuse and chondrify along the midline [15]. By the 10th week of development, a cartilaginous midline structure has been formed, called the sternal plate [6]. The sternal plate consists of six segmental sternebrae that ultimately delineate into three articulating segments through sternebrae fusion. Eventually, the cranial sternebra forms the manubrium, the four intermediate sternebrae collectively form the body of the sternum, and the caudal sternebra forms the xiphoid process [1,5]. Ossification centers arise craniocaudally, beginning in the manubrium of the sternum and the upper part of the body of the sternum at 19 weeks gestation [5]. By the time of birth, the manubrium and the body of the sternum are partially ossified, while the xiphoid process remains completely cartilaginous [9].

The primary ossification center of the xiphoid process does not appear until the 1st decade of life [6]. Subsequent ossification begins around six years of age [6] and can take as long as four to five decades to complete [2]. As noted above, there are even reported instances of ossification that have been delayed until the 8th decade of life [10]. This prolonged developmental period increases the likelihood of sternal variations [6], including incomplete ossification of the xiphoid process and/or xiphoid process foramina [16]. In a study analyzing 1,000 subjects for sternal anomalies, multidetector computed tomography (MDCT) imaging failed to detect any degree of xiphoid ossification in 23 subjects, with the majority of these failures (n=610, 61%) in those < 30 years old [16]. Partial xiphoid ossification was detected in 48 subjects, or 4.8% [16]. Using a similar MDCT methodology to evaluate variations of the xiphoid process among 500 adults (age range: 18 to 90 y/o), partial xiphoid ossification was detected and classified as the superior 1/3 portion (10.6%), superior 2/3 portion (36.4%), and the middle 1/3 portion (1%) [10]. Such large variances between the reported results of the aforementioned studies may contribute to the medical community’s sporadic awareness of xiphoid variations. 

Most interestingly, visual observation of the presented case best aligns with a partial ossification of the superior 2/3 of the xiphoid process, a potentially rare anatomic finding given the age of our elderly (75-year-old) donor. In addition to the noted partial ossification, the presented case also contained a xiphoid process foramen. Interestingly, the xiphoid process foramina is the least likely sternal foramina to form. In a meta-analysis of 33 studies with 15,223 subjects, the pooled prevalence of sternal body and/or xiphoid foramina combined was only 8.9% [17]. There was a noted sex-specific difference, with sternal foramina being more common among males (12.2%) than females (6.8%) [17]. Moreover, the presence of a foramen was more frequently encountered in South American and African populations compared to North American and European populations, but the literature does not report a clear consensus on this [17,18]. When sternal foramina are discovered, they are most likely located in the lower sternal body (78.8% of the time), with extremely rare cases in the manubrium of the sternum [9,19]. Although there have been documented cases of foramina in the manubrium, none were encountered in a study screening 1,000 patients for sternal variations [16]. Most applicable to this case report, a meta-analysis of 33 studies investigating foramina throughout the sternum within the general population found the prevalence of a xiphoid process foramen was only 2.9% [17]. However, in the same meta-analysis, a sub-analysis of three studies that only examined the xiphoid process for the presence of potential foramina indicated a pooled prevalence of foramina of 51.9% [17]. A potential explanation for this discrepancy proposed by the authors was that the xiphoid process may be inadvertently overlooked in studies that aim to examine the entire sternum [17]. Additionally, the discrepancy in prevalence rates may be directly linked to the age of the population studied, as we know the xiphoid process commonly takes decades to fully ossify [9]. Further research is required to validate such speculations and further elucidate our understanding of the xiphoid process foramina. 

Although the xiphoid process is the smallest of the three sternal elements, the literature reports the highest degree of variation in its shape, including broad and thin, pointed, bifid, trifid, curved, deflected, or containing foramina [1,3,11]. In a recent large-scale report which assessed the xiphoid processes of 41 cadavers via radiography and 902 patients via MDCT, the morphology of the human xiphoid process was classified by examining three shape-based categories: anatomic shape, presence or absence of foramina, and (if present) characteristics of such foramina [20]. First, xiphoid processes were classified by their overall shape into one of three categories: *type I* was oval-shaped, *type II *was inferiorly pointed, and *type III* was inferiorly forked [20]. Second, xiphoid processes were named *type a* if no foramen was present and *type b* if one or more foramina were present. If present, the foramina themselves were classified into one of four categories based on size and distribution: *type L* had a single large foramen of a diameter greater than 5 mm; *type S* had a single small foramen of a diameter less than 5 mm; *type LS* had both a large and a small foramen; *type SS* had two or more small foramina [20]. The most common xiphoid foramen was a *type L*, observed in 55.51% of type b subjects [20]. 

Our recorded measurements of the sternum, the xiphoid process, and its foramen are similar in average length and maximum width to other reported cases of a teardrop- or pear-shaped xiphoid foramen [4,20]. Using the previously described classification system, the presented clinical case can be categorized as a *type Ib* (oval-shaped, with a foramen) xiphoid process with a *type L* (single, larger than 0.5cm) xiphoid foramen [20]. 

Although minor xiphoid process variations are relatively common and often asymptomatic for the patient, medical professionals and acupuncturists must be aware of them for appropriate patient care. Specifically, the potential presence of a xiphoid process foramen is important for differential diagnosis between the anatomical structure itself and other pathologies, as this variation may easily be misdiagnosed during bone scintigraphy for traumatic injury to the area or for lytic lesions, such as cysts, granulomas, sarcoma, or metastatic lesions [6,12,18]. Moreover, the xiphoid process foramen itself poses the risk of injury to nearby underlying mediastinal structures during medical interventions [6,18]. For example, unexpected xiphoid foramina at the level of an acupuncture point has resulted in 14 recorded cases of cardiac tamponade from acupuncture treatments [18]. Therefore, during thoracic or acupunctural interventions, healthcare professionals must have appropriate thoracic anatomical knowledge, as well as a thorough understanding of patients’ computed tomography scans, to minimize the risk of unintentionally puncturing thoracic structures such as the heart or lungs [18]. 

In addition to the overall shape of the xiphoid process and features of the xiphoid foramen, the anterior deviation of the presented xiphoid process makes this clinical case unique to a further degree. A typical xiphisternal angle has a slight anterior inclination that measures within a range of 140° to 199°; a value < 140° indicates an anterior deviation of the xiphoid process and may contribute to the inflammation and discomfort associated with xiphodynia [13]. In a study analyzing the xiphoid process of 500 adults free of any xiphoid process diseases through MDCT, an anteriorly deviated xiphoid process was present in 65.4% of subjects [3]. However, another MDCT study reported that straight alignment was the most common xiphoid process presentation (54.5%), followed by anterior deviation (23.0%), dorsal deviation (20.3%), and then s-shaped deviations (2.2%) [20]. Albeit rare, anterior deviation of the xiphoid process can induce xiphodynia, where patients experience pain while breathing, lying in a prone position, or applying light pressure to the anterior prominence [13]. Often, this condition is triggered by trauma to the anterior thoracic wall and can be mistaken for pain originating in other thoraco-abdominal organs [13]. 

While there is a significant anterior deviation of the xiphoid process in the presented clinical case, we are unable to confirm or refute a xiphodynia diagnosis, as this would require the manifestation of clinical symptoms as well. To uphold the integrity of the cadaver’s confidentiality, we did not seek to further explore the donor’s medical history beyond what is provided at the time of body donation. Therefore, we are unable to speculate on potential patient symptoms. However, the presented clinical case’s objective measurements and qualitative observations of the anteriorly deviated xiphoid process resemble those of previous case reports [13], making it reasonable to hypothesize that xiphodynia may have been experienced by our donor during his lifetime.

## Conclusions

This case report presents three rare sternal variations discovered during the educational dissection of a male human donor: a partially ossified, anteriorly deviated xiphoid process that contains a large teardrop-shaped xiphoid foramen. To the best of our knowledge, all three of these xiphoid variations have not been previously reported within the same patient. Thus, the presented findings are extraordinary. Despite minor xiphoid process variations being relatively common among patients, the xiphoid process foramen is less reported. Due to its proximity to underlying thoracic and abdominal structures, medical professionals and acupuncturists risk adverse procedural complications if they do not have thorough anatomical knowledge. Therefore, a comprehensive understanding of the xiphoid process’s possible variations can play a significant role in preventing misdiagnoses or unforeseen complications.
